# The role of venoactive compounds in the treatment of chronic venous disease

**DOI:** 10.1016/j.jvsv.2025.102258

**Published:** 2025-05-08

**Authors:** Monika Lecomte Gloviczki, Stavros K. Kakkos, Tomasz Urbanek, John Chuback, Andrew Nicolaides

**Affiliations:** a Department of Internal Medicine and Gonda Vascular Center, Mayo Clinic, Rochester, MN; b Vascular Science & Art, LLC, Scottsdale, AZ; c Department of Vascular Surgery, University of Patras Medical School, Patras, Greece; d Department of General Surgery, Vascular Surgery, Angiology and Phlebology, Medical University of Silesia, Katowice, Poland; e Chuback Vein Center, Paramus, NJ; f Vascular Screening and Diagnostic Centre, Nicosia, Cyprus; g Imperial College, London, UK; h University of Nicosia Medical School, Nicosia, Cyprus

**Keywords:** Chronic venous disease, Venoactive compounds or drugs, Calcium dobesilate, Diosmin, Hidrosmin, Horse chestnut seed extract, Hydroxyethylrutosides, Micronized purified flavonoid fraction, Pentoxifylline, Red vine leaf extract, Ruscus extract, Sulodexide, Venous symptoms, Venous edema, Venous leg ulcers

## Abstract

**Background:**

Chronic venous disease (CVD) is a major global health issue, affecting millions of people and contributing to significant morbidity and economic strain. The condition's pathophysiology is complex, involving both mechanical and biochemical processes that lead to venous reflux, obstruction, and chronic inflammation. This review focuses on the role of venoactive compounds (VACs), also known as venoactive drugs in Europe and other parts of the world, in managing CVD. The aim was to review the scientific evidence and to define the role of VACs within the comprehensive treatment algorithm for CVD, alongside established and well adopted interventional therapies and noninterventional therapies such as compression.

**Methods:**

The review of the scientific evidence was done on VACs mechanism of action and efficacy in alleviating CVD symptoms, reducing swelling or venous edema, and improving healing of venous leg ulcers. Whenever available, systematic reviews, meta-analyses and randomized controlled trials were used. The quality of evidence assessment followed the GRADE methodology from A (high), B (moderate), to C (low to very low) quality.

**Results:**

Venoactive drugs or compounds share similar effects, such as sealing the endothelial barrier, enhancing lymphatic drainage, reducing edema, improving venous tone, inhibiting leukocyte adhesion to vein walls/valves and inflammatory mediator release, lowering blood viscosity, and promoting red blood cell flexibility. Scientific evidence on the VACs effectiveness on CVD symptoms (pain, cramps, and heaviness) and swelling or edema have shown some variability. Micronized purified flavonoid fraction (MPFF) and Ruscus extract combined with hesperidin methyl chalcone and ascorbic acid had the highest, mostly level A, quality of evidence. In venous leg ulcers, micronized purified flavonoid fraction, sulodexide, and pentoxifylline were the most effective adjunctive treatments, with evidence level A.

**Conclusions:**

The existing scientific evidence provides a strong rationale for incorporating VACs into a comprehensive treatment plan for CVD, alongside established interventional therapies and noninterventional approaches like compression, to optimize patient outcomes and improve quality of life.

Chronic venous disease (CVD) represents a significant global health concern, affecting millions of individuals and leading to considerable morbidity and economic burden.^1^, ^2^, ^3^ CVD results from the progressive dysfunction of venous structures and venous hypertension.^4^ Symptoms are ranging from mild discomfort to severe complications such as venous ulcers and deep vein thrombosis. The underlying pathophysiology is multifactorial, with mechanical and biochemical processes that culminate in venous reflux, venous obstruction, and chronic inflammation.^5^^,^^6^ This inflammation is pivotal, because it perpetuates tissue damage and exacerbates the clinical symptoms experienced by patients.^6^^,^^7^

Several well-established risk factors contribute to the development of CVD, including advanced age, obesity, sedentary lifestyle, and a history of pregnancy or hormonal therapy.^8^^,^^9^ Genetics play a crucial role, with a strong familial component observed in many cases of CVD.^10^^,^^11^ The CEAP classification system—an acronym for Clinical, Etiological, Anatomic, and Pathophysiological—serves as a standardized framework for grading CVD.^12^^,^^13^ This system ranges from CEAP class C0 (no visible signs) to C6 (active venous ulcer), allowing clinicians to assess and communicate the stage of the disease.

In addition to clinical classification, the Venous Clinical Severity Score offers an evaluation of CVD severity.^13^ The Venous Clinical Severity Score quantifies clinical symptoms such as pain, swelling, and the presence of venous ulcers, providing a comprehensive scoring system to monitor disease progression and treatment efficacy.

The main body of this article delves into the critical yet often underappreciated role that venoactive compounds (VACs) or venoactive drugs, a term used in Europe and other parts of the world for some of them, play in the management of CVD. These compounds have demonstrated efficacy in alleviating symptoms associated with CVD and improving venous leg ulcer (VLU) healing.^14^^,^^15^ In addition to pharmacological interventions, other therapies are effective in treating CVD.^15^, ^16^, ^17^ Techniques such as thermal venous ablation, vein stripping and microphlebectomy, and nonthermal venous ablations using foam sclerotherapy and glue sealant are integral components of a comprehensive treatment strategy.

As research continues to elucidate the VACs' mechanism of action, it is clear that these agents provide symptomatic relief for pain or aching, cramps, heaviness, and fatigue.^14^ Additionally, VACs help to reduce and prevent lower extremity edema and, to a certain extent, improve associated chronic skin changes. Perhaps most important, some VACs have also been proven to accelerate and enhance the healing process of VLUs, one of the most challenging complications of CVD.^14^^,^^18^^,^^19^ This article aimed to highlight the importance of integrating VACs into the comprehensive treatment algorithm for CVD, alongside established and well-adopted interventional therapies and noninterventional therapies such as compression therapy, to optimize patient outcomes and improve quality of life (QoL). The authors believe that VACs in the United States are under-recognized, poorly understood, and largely dismissed by clinicians in the contemporary care of the patient with CVD. We aim to provide evidence that VACs should be more widely much more widely incorporated into daily treatment regimens.

## Mechanism of action of different VACS

Pharmacological therapy together with the lifestyle changes, risk factor elimination, compression, and other treatment modalities, remains one of the important measures in the symptomatic CVD management.^14^^,^^15^ According to the Vein Glossary published in 2020^20^ venoactive drugs are “a heterogeneous group of plant-derived, animal-derived, or synthetic medicinal products that have effects on oedema and symptoms associated with chronic venous disorders.” Furthermore, the authors note that venoactive drugs share similar effects, such as sealing the endothelial barrier, enhancing lymphatic drainage, reducing orthostatic edema, improving venous tone, inhibiting leukocyte adhesion to vein walls/valves and inflammatory mediator release, lowering blood viscosity, and promoting red blood cell flexibility. VACs may be used as monotherapy or in combination with other molecules (eg, micronized purified flavonoid fraction [MPFF], combination of Ruscus extract with hesperidin methyl chalcone [HMC] and ascorbic acid [AA]). The principal groups of VACs with their representatives are presented in the Table I.

**Table I:** Categories of the venoactive compounds (VACs) (modified from Ramelet et al^21^), their presence in the US and dosages

Category	Venoactive drugs	Presence in the US as nutritional supplement	Dosage (mg/day)
Plant derived			
Gamma-benzopyrones (flavonoids)	MPFF	Yes	1000
	Diosmin	Yes	300-600
	Rutin and its derivatives, O-(beta-hydroxyethyl)-rutosides	Yes	1000
	Kaempferol glucoside, Quercetin glucuronide	Yes	100-300
Alpha-benzopyrones	Coumarin	Yes	90, included in some anti-edema supplements
Saponins	Ruscus extract	Yes	300
	HCSE, Escin	Yes	Initially 120, then 60
Other plant extracts	Ginko Biloba extracts	Yes	125
	Proanthocyanidols	Yes	300-360
	Anthocyanosides	Yes	100-300
Chemical synthesis			
Synthetic products	Calcium dobesilate	Yes, as a prescription medication	1000-1500
	Naftazone	No	30
	Benzarone	No	400-600
Animal products derived			
Glycosaminoglycans	Sulodexide	No	VLU treatment: 600 LSU once daily for 20 days followed by 2 ×500 LSU orally. Symptomatic CVD treatment: 2× 500 LSU oral route

*CVD,* Chronic venous disease; *HCSE,* horse chestnut seed extract; *LSU,* lipasemic units; *MPFF,* micronized purified flavonoid fraction; *VLU,* venous leg ulcer.

One of the most important proprieties of VACs is venous tone increase.^14^^,^^15^ This can be related to effects on norepinephrine metabolism (MPFF), agonist action on the alfa-1 adrenergic receptors (Ruscus extract + HMC + AA), calcium ions related contraction activity (horse chestnut seed extract [HCSE]) or metalloproteinase expression and metabolism (sulodexide).^14^^,^^22^ Various other actions should be mentioned, including inflammation inhibition, endothelial cell protection, and free radical generation decrease, as well as lymphatic drainage increase.^14^^,^^21^^,^^23^^,^^24^ When describing the potential anti-inflammatory effects, different stages of inflammation can be modulated or downregulated from the leucocytes' adhesion molecules expression and endothelial cell activation to the inflammatory cell extravasation, oxygen free radical production, and metalloproteinase expression and activation with growth factors release.^14^^,^^23^ Among the mechanisms related to the VACs’ effect on edema, a sealing of the endothelial barrier has been documented in several trials concerning MPFF, Ruscus, rutosides, escin, calcium dobesilate or sulodexide.^14^^,^^24^ Another important factor leading to the venous edema reduction is lymphatic drainage increase, proved in the research on MPFF, Ruscus, calcium dobesilate, or alfa benzopyrones.^14^^,^^25^

Several differences between VACs can be noticed and not all compounds classified as venoactive work in the same way; furthermore, not all of them show activity in all potentially beneficial directions (Table II).^14^ For some, additional proprieties have been identified, such as glycocalyx layer restoration or antithrombotic activity of sulodexide.^26^ The lack of the VACs group homogeneity in terms of their biological and molecular characteristics corresponds with the potential differences in their clinical efficacy as well.^14^

**Table II:** Mode of action of venoactive compounds (VACs) (modified based on references Nicolaides et al,^14^ Raffeto et al,^22^ Nicolaides et al,^23^^,^^24^ Monjotin et al,^25^ and Carroll et al^26^)

	Effects on venous tone	Effect on changes in the vessel wall and venous valves	Effect on the sealing of the endothelial barrier in microcirculation (endothelial barrier permeability decrease)	Improvement of lymphatic drainage	Hemorheological abnormalities improvement	Effect on the generation of oxygen free radicals
Flavonoids						
MPFF	+	+	+	+	+	+
Rutin and rutosides	+		+	+	+	+
Anthocyanins/red vine leaf extract			+			+
Proanthocyanidins/red vine leaf extract/						+
Saponins						
HCSE, Escin	+		+			+
Ruscus extract	+	+	+	+	+	+
Synthetic products						
Calcium dobesilate	+		+	+	+	+
Glycosaminoglycans						
Sulodexide	+	+	+		+	+

*HCSE,* Horse chestnut seed extract; *MPFF,* micronized purified flavonoid fraction.

## Scientific evidence on clinical efficacy of VACS in CVD

### Symptoms

CVD may cause a number of leg symptoms, fully described by the SYM vein consensus document.^27^ Meta-analyses on the effectiveness of most VACs, presented below, have shown that they can improve some of these symptoms. The relative efficacy of the various VACs is variable and not all of them are effective for a particular symptom,^14^^,^^28^ as demonstrated by randomized controlled trials (RCTs) and meta-analyses, including a Cochrane review.^29^^,^^30^ This review confirmed that venoactive drugs have beneficial effects on symptoms with some adverse effects for calcium dobesilate and hydroxyethylrutosides (HRs), mostly minor and usually gastrointestinal (Table III).^30^ Regarding clinical efficacy more specifically, the same review reported that pain was reduced with aminaftone, calcium dobesilate, French maritime pine bark extract, and HR, as well as diosmin and hidrosmin considered as a group. Cramps were reduced with aminaftone, calcium dobesilate, HR, as well as diosmin and hidrosmine considered as a group. Restless leg syndrome symptoms were reduced only with calcium dobesilate. Pruritus was reduced with aminaftone and HR. Leg heaviness was reduced with aminaftone, French maritime pine bark extract, and HR, as well as diosmin and hidrosmin considered as a group. Swelling was reduced with calcium dobesilate, French maritime pine bark extract, and HR, as well as also diosmin and hidrosmin considered as a group. HR was the only VAC to reduce paresthesia.

**Table III:** Adverse events of the most commonly used venoactive compounds (VACs)

	Adverse events	Rates (%) vs placebo
MPFF^30^^,^^31^	Gastrointestinal disorders (diarrhea, indigestion, nausea and vomiting), dizziness, headache, discomfort, rash, and itching	10-15.9 vs 13.9 (NS)
Diosmin and hidrosmin^30^^,^^31^	Gastrointestinal disorders (heartburn and nausea)	11.36-13.75 vs 14.95 (NS)
HR^30^^,^^32^	Gastrointestinal disorders (abdominal pain, constipation, diarrhea, dry mouth, epigastric discomfort, flatulence, nausea, vomiting), headache	20 vs 16 (NS)
Red vine leaf extract^30^^,^^33^	Constipation, gastralgia, headache, allergic reaction	11 vs 20
Ruscus extract + HMC + AA^30^^,^^34^	Acidity, epigastralgia, diarrhea	7.89
HCSE^24^^,^^35^	Mild and infrequent; gastrointestinal complaints, dizziness, nausea, headache and pruritus	1-36
Sulodexide^36^	Cutaneous rash, diarrhea, epigastric pain, and headache	17.14 vs 13.07 (NS)
Calcium dobesilate^24^^,^^30^^,^^37^^,^^38^	Gastrointestinal events (epigastric discomfort, vomiting), agranulocytosis (prevalence lesser than in the general population)	9.7 vs 7.6 (NS)

*HCSE,* Horse chestnut seed extract; *HR,* hydroxyethylrutosides; *MPFF,* micronized purified flavonoid fraction; *NS,* nonsignificant; *Ruscus Extract + HMC + AA,* Ruscus extract combined with hesperidin methyl chalcone (HMC) and ascorbic acid (AA).

#### MPFF

A systematic review and meta-analysis of seven double-blind placebo-controlled RCTs on 1692 patients with CVD, studied the effectiveness of MPFF across the spectrum of defined venous symptoms, QoL, and treatment assessment by the physician.^39^ On qualitative analysis, MPFF, compared with placebo, significantly improved nine defined leg symptoms, including pain, heaviness, feeling of swelling, cramps, paresthesia, burning sensation, and pruritus, as well as functional discomfort and QoL. On quantitative analysis, MPFF compared with placebo, assessed as a categorical variable, showed a statistically significant reduction of leg pain (risk ratio [RR], 0.53; number needed to treat [NNT], =4.2), heaviness (RR, 0.35; NNT, 2.0), feeling of swelling (RR, 0.39; NNT, 3.1), cramps (RR, 0.51; NNT, 4.8), paresthesia (RR, 0.45; NNT, 3.5), and functional discomfort (RR, 0.41; NNT, 3.0). Similarly, MPFF compared with placebo, assessed as a continuous variable showed a statistically significant reduction of pain (standardized mean difference [SMD], −0.25), heaviness (SMD, −0.80), feeling of swelling (SMD, −0.99), burning sensation (SMD, −0.46), cramps (SMD, −0.46), and functional discomfort (SMD, −0.87). Regarding objective assessments of leg edema, the use of MPFF compared with placebo showed a statistically significant clinical improvement as assessed by the physician (RR, 0.28; NNT, 2.5) and also improvement of QoL (SMD, −0.21). Heterogeneity was mostly minimal, although the existing evidence, where sufficient, was mostly of high certainty, indicating that further research is very unlikely to change the confidence in the estimate of effect. Real-world studies, such as VEIN STEP (Chronic VEnous dIsorders maNagement and Treatment effectivenesS evaluaTion in Chronic vEnous Disease, an International Program) have confirmed the clinical effectiveness of MPFF.^40^ In an emerging area to improve patient symptoms after intervention, a beneficial effect has been shown and acknowledged by the guidelines.^14^^,^^17^

Diosmin has limited information from RCTs, perhaps because, for the last 30 years, MPFF as an improved form is widely available with better bioavailability owing to improved gastrointestinal absorption,^41^ better clinical results, and reduced gastrointestinal side effects.^31^ A reanalysis of the difference in clinical effectiveness for MPFF and diosmin by Cospite and Dominici^31^ shows a superiority of MPFF for the outcome measures of leg heaviness (*P* = .007), sensation of swelling (*P* = .0058) and burning sensation (*P* = .030) at day 30, with results maintained at day 60 (*P* = .007, *P* = .0007, and *P* = .030), respectively, and also superiority of MPFF for the leg pain improvement at day 60 (*P* = .0058).

Diosmin preparations are still available, although real-world evidence shows a lesser efficacy for the symptoms of pain (*P* = .002), leg heaviness (*P* < .001), and sensation of swelling (*P* = .008), and also for QoL (*P* < .001), compared with MPFF.^40^

#### HRs

A systematic review and meta-analysis of 15 trials involving 1643 patients studied the effectiveness of HR for improvement of the symptoms of CVD.^32^ These showed that HR significantly reduced symptoms of pain (SMD, −1.07) and cramps (SMD, −1.07) when assessed as a continuous variable, and also leg heaviness (odds ratio, 0.50). However, the authors acknowledge that not all results could be entered in the meta-analysis because of heterogeneity in reporting, and the moderate quality of the RCTs because of unclear risk of bias in the allocation concealment and other domains.

#### Red vine leaf extract

Red vine leaf extract was evaluated in two double-blind, placebo-controlled trial RCTs involving a total of 510 patients.^33^^,^^42^ Red vine leaf extract significantly reduced CVD-related symptoms vs placebo; more specifically, the first trial randomized 260 patients and showed a highly significant decrease in the intensity of leg heaviness, tightness, paresthesiae, and pain.^42^ It is important to note that only pain reduction was significant at 6 weeks and that the other symptoms were significantly reduced at the 12-week follow-up. The second study randomized 250 patients.^33^ Leg heaviness improved but not significantly, tightness was significantly reduced on day 42, and pain improved significantly on day 84.

#### Ruscus extract combined with HMC and AA

A systematic review and meta-analysis of 10 double-blind placebo-controlled RCTs involving 719 patients (CEAP clinical class ranging between 2 and 5) studied the effectiveness of Ruscus extracts + HMC + AA.^34^ On qualitative analysis, Ruscus + HMC + AA significantly improved pain, heaviness, fatigue, feeling of swelling, cramps, itching and paresthesia. On quantitative analysis, Ruscus + HMC + AA compared with placebo, for symptoms assessed as a categorical variable, showed a statistically significant reduction of leg pain (RR, 0.35; NNT, 5), heaviness (RR, 0.26; NNT, 2.4), feeling of swelling (RR, 0.53; NNT, 4), paresthesia (RR, 0.27; NNT, 1.8), global symptoms (RR, 0.54; NNT, 4.3), and the total number of venous symptoms (RR, 0.41). Similarly, when symptoms were assessed as a continuous variable, there was a statistically significant reduction of pain (SMD, −0.80), heaviness (SMD, −1.23), fatigue (SMD, −1.16), feeling of swelling (SMD, −2.27), and paresthesia (SMD, −0.86). The existing evidence was mostly of high quality.

#### HCSE

A Cochrane review on HCSE identified 17 RCTs involving 1593 patients.^35^ Ten RCTs were placebo controlled, two RCTs compared HCSE with compression stockings and placebo, and five compared HCSE with another VAC. Leg pain was assessed in seven RCTs, with six studies (n = 543) reporting a statistically significant reduction in leg pain with HCSE vs placebo. Pruritus was assessed in eight placebo-controlled RCTs, with four of them (n = 407) showing a statistically significant reduction with HCSE vs placebo and two of them showing a statistically significant reduction compared with baseline. Edema was assessed in six placebo-controlled RCTs, with four of them (n = 461) showing a statistically significant reduction vs placebo, and one of them showing an improvement compared with baseline.

#### Sulodexide

A systematic literature review and network meta-analysis of sulodexide identified 11 observational studies with 1267 patients, assessing pain and feeling of swelling, heaviness, cramps, and paresthesiae.^19^ The overall effect estimates from the meta-analyses showed that sulodexide significantly decreased pain by −1.60 score points (95% confidence interval [CI], −2.47 to −0.72] on a 4-point Likert scale (I^2^ = 86%, *P* < .01) and the feeling of swelling by −1.64 score points ( 95% CI, −1.90 to −1.38). Sulodexide was also effective in significantly decreasing heaviness by a −1.56-point score (95% CI, −1.87 to−1.25) and paresthesiae by a −1.54-point score (95% CI, −2.21 to−0.83) on Likert scales, with zero heterogeneity and high heterogeneity (I^2^ = 82%), respectively. A previous meta-analysis of 13 observational studies including 1901 patients reported that sulodexide significantly decreased the intensity of pain, cramps, heaviness, paresthesiae and total symptom score.^36^

#### Calcium dobesilate

A 2004 systematic review and meta-analysis of 10 double-blind placebo-controlled RCTs on 778 patients studied the effectiveness of calcium dobesilate.^37^ On quantitative analysis, calcium dobesilate significantly improved night cramps (NNT, 8; 95% CI, 4-50) and discomfort (NNT, 4; 95% CI, 3-7). On qualitative analysis, there was also improvement of the outcome measures of pain, lower limb heaviness, edema, and investigator's opinion of symptom improvement. Because of statistically significant heterogeneity, their results were not pooled. A subgroup analysis showed greater improvements in pain, heaviness, and paresthesia in the group of patients with severe symptoms than in those with mild symptoms. On meta-regression analysis, greater efficacy of dobesilate was correlated with greater disease severity. During the last 20 years, an additional four double-blind placebo-controlled RCTs were conducted and included 1165 patients.^38^^,^^43^, ^44^, ^45^ These reported that calcium dobesilate improved pain,^43^, ^44^, ^45^ QoL,^38^ and discomfort.^44^^,^^45^

#### Interpretation of evidence on VACs efficacy on venous symptoms

The results of the studies presented have been the background for the recommendations made by the various guidelines.^14^^,^^15^^,^^17^^,^^28^ The relative efficacy of the most commonly used VACs, according to the European Venous Forum/International Union of Angiology guidelines is shown in Table IV.^14^^,^^28^ It is clear that the two VACs with the most scientific evidence are MPFF and Ruscus extracts, and that there are relative differences in the efficacy of the various VACs. Using the Cohen classification that defined a SMD of 0.8 or greater as large,^46^ the reader can realize that the effect of VACs on several symptoms shown in the Table III is clinically significant, fact that is also supported by a very low NNT. Furthermore, these two VACs are the only ones with multiple symptoms being awarded a high certainty of evidence according to the GRADE system.^34^^,^^39^ A similar distinction was recently made by the 2023 Society for Vascular Surgery, American Venous Forum, and American Vein and Lymphatic Society clinical practice guidelines for the management of varicose veins of the lower extremities, in favor of MPFF and Ruscus extracts, which have been studied more extensively in double-blind, placebo-controlled RCTs and meta-analyses.^17^ For patients with symptomatic CVD, who are not undergoing interventional treatment, are awaiting intervention, or have persisting symptoms after intervention, the European Society for Vascular Surgery CVD guidelines suggest that based on the available evidence for each individual drug, medical treatment with venoactive drugs should be considered.^15^

**Table IV:** Relative efficacy of the most commonly used venoactive compounds (VACs)

Symptom	MPFF	Ruscus + HMC + AA	HR	HCSE	Calcium dobesilate
Pain (NNT)	A (4.2)	A (5)	B	A (5.1)	B (1.4)
SMD	−0.25	−0.80	−1.07		
Heaviness (NNT)	A (2.9)	A (2.4)	B (17)		A (1)
SMD	−0.80	−1.23	−1.00		
Functional discomfort/discomfort (NNT)	A (3)				B (4)
SMD	−0.87				
Leg fatigue (NNT)	NS	B			
SMD		−1.16			
Cramps (NNT)	B (4.8)	B/C	B		
SMD	−0.46		−1.7		
Paresthesiae (NNT)	B/C (3.5)	A (1.8)			B (2)
SMD	−0.11	−0.86			
Burning (NNT)	B/C	NS			
SMD	−0.46				
Pruritus/itching (NNT)		B/C		A (6.1)	
Tightness (NNT)	NS				
Restless legs (NNT)	NS				
QoL	A				NS
SMD	−0.21				

*AA,* Ascorbic acid; *HMC,* hesperidin methyl chalcone; *HCSE,* horse chestnut extract; *HR,* hydroxyethylrutosides; *MPFF,* micronized purified flavonoid fraction; *NNT,* number needed to treat; *NS,* not significant; *QoL,* quality of life; *SMD,* standardized mean difference.

Level of evidence that merits grade A or B for the effect of the main venoactive drugs on individual symptoms and QoL with magnitude of effect: NNT to benefit one patient or SMD are also shown. Only randomized placebo-controlled trials and meta-analyses were considered.

Modified from Nicolaides et al, with permission from the publisher.

Feeling or sensation of swelling is presented in the Table IV with the edema.

### Swelling and edema

A 2020 Cochrane review including 69 RCTs^30^ reported only moderate-certainty evidence that VACs reduce edema in the lower legs compared with placebo (RR, 0.70). However, meta-analyses and RTCs for individual VACs provided high-quality evidence of benefit for some compounds.

A meta-analysis of 10 calcium dobesilate RCTs found that reductions in malleolar swelling and leg volume were more pronounced in severe disease compared with those with mild disease.^37^ Another meta-analysis comparing results of calcium dobesilate RCTs with several VACs found it to be the most effective in reducing foot volume (mean reduction of −85 cm^3^/mL vs placebo).^19^

For diosmin, a review of three RCTs found that its effects on the sensation of leg swelling are comparable with MPFF,^47^ in contrast with a meta-analysis by Allaert.^48^

A Cochrane review analyzed seven HCSE RCTs vs placebo.^35^ The HCSE antiedema effect, with weighted mean difference in leg volume reduction of 32.1 mL, was confirmed in six of seven trials. One trial reported comparable average leg volume reductions between the HCSE group and the compression group, with decreases of 43.8 mL and 46.7 mL, respectively.^35^^,^^49^

A meta-analysis conducted by Pompilio et al^19^ showed for HRs the greatest improvement in the mean swelling sensation score. In the study by Allaert,^48^ the mean reduction in ankle circumference with HR was –0.58 ± 0.31 cm (*P* < .0001 vs placebo), comparable with Ruscus, but inferior to MPFF.

A meta-analysis by Kakkos^39^ for MPFF included seven high-quality RCTs vs placebo in CVD. The assessment of feeling of swelling showed a SMD of −0.99 (continuous variable), and RR of 0.39 (categorical variable). The NNT was 3.1. For the effect on ankle circumference the authors reported moderate quality of evidence, with SMD −0.59. Another meta-analysis of 10 RCTs vs placebo compared the effects of MPFF, HR, Ruscus, and diosmin on venous edema assessed by ankle circumference.^48^ MPFF demonstrated the highest efficacy in reducing edema (*P* < .00,001 vs placebo and other compounds). A Bayesian network analysis of 45 RCTs and 18 observational studies evaluated the efficacy of MPFF compared with other compounds: calcium dobesilate, HCSE, HR, Ruscus extract, pentoxifylline, and sulodexide.^19^ MPFF was found to be the most effective VAC in improving leg volume. Water plethysmography was used in a RCT in venous edema that included 136 patients assigned to four treatment groups: MPFF, aminaphtone, coumarin + troxerutin, and placebo.^50^ The MPFF group showed the highest frequency of volume reduction 100 mL or more.

A systematic review of five trials evaluated red vine leaf extract effects on edema.^51^ Although significant improvements were reported in two trials,^33^^,^^52^ the results were not consistent across all studies.

A meta-analysis of Ruscus extract + HMC + AA involving 25 RCTs and 6 observational studies^53^ did not reach a definitive conclusion regarding the compound's anti-edema efficacy. However, another meta-analysis of 10 RCTs^34^ found that Ruscus + HMC + AA, compared with placebo, significantly reduced the sensation of swelling (RR, 0.53; *P* < .0001, NNT = 4), decreased ankle circumference and leg/foot volume. Ruscus extract + HMC + AA showed the greatest effectiveness in reducing ankle circumference among VACs in one comparative meta-analysis,^19^ and it ranked second best after MPFF in another.^48^

A meta-analysis of 64 studies, which included 13 trials (1901 CVD patients), found sulodexide effective in reducing edema.^36^ Another meta-analysis, involving observational studies of CVD patients without a VLU, showed a significant improvement in the sensation of swelling.^19^

A summary of the levels of scientific evidence for efficacy on sensation of swelling and edema is provided in Table V.

**Table V:** Efficacy of the most commonly used venoactive compounds (VACs) in swelling and edema reduction

	MPFF	Ruscus + HMC + AA	HR	HCSE	Calcium dobesilate	Sulodexide
Sensation of swelling (NNT)	A (3.1)	A (4)	A	A	A	B
SMD	−0.99	−2.27				
Edema	A SMD −0.59	A	A Mean reduction in ankle circumference −0.58 ± 0.31 cm	A WMD −32.1 mL	A −85 cm^3^/mL vs placebo	B

*AA,* Ascorbic acid; *HMC,* hesperidin methyl chalcone; *HCSE,* horse chestnut extract; *HR,* hydroxyethylrutosides; *MPFF,* micronized purified flavonoid fraction; *NNT,* number needed to treat; *SMD,* standardized mean difference; *WMD,* weighted mean difference.

Level of evidence that merits grade A or B for the effect of the main VACs on sensation of swelling and edema with magnitude of effect: NNT to benefit one patient or SMD are also shown. Only randomized placebo-controlled trials and meta-analyses were considered.

### Ulcers and skin changes

Venous leg ulceration, being the advanced stage of CVD, requires complex and disease-oriented treatment. One of the main goals of the treatment is venous hypertension decrease, which can be lowered using medical compression and (if feasible) venous reflux ablation or deep vein obstruction treatment. Venous hypertension treatment in VLU patients should be combined with the proper local wound management and can be supported by the adjunctive medical therapy based on the compounds with documented evidence in this indication. According to the 2018 European Venous Forum Guidelines, three drugs with the highest level of evidence in VLU healing can be specified: pentoxifylline, MPFF, and sulodexide.^14^ The 2022 European Society for Vascular Surgery guidelines suggest the use of MPFF, HRs, pentoxifylline, or sulodexide as an adjunct treatment in the patients with a VLU.^15^

The level of scientific evidence positions some of the VACs in the armamentarium of the VLU medical therapies (Table VI).^14^^,^^15^ The biological proprieties of the VACs interfere with several pathological processes related to the venous hypertension and VLU development.^14^ The presence of inflammation, free radical generation, vessel hyperpermeability, impaired lymphatic drainage, metalloproteinase expression, and activation reported in the patients with a VLU, can be potentially modulated and downregulated by the medical therapy. However, despite the wide theoretical background of this approach, only some of the VACs confirmed their clinical efficacy in VLU treatment, supported in good-quality clinical trials.^14^

**Table VI:** Efficacy of the most commonly used venoactive compounds (VACs) and pentoxifylline in venous leg ulcers (*VLUs*)

	MPFF	HR	Pentoxifylline	Sulodexide
Skin changes	C	–	–	C
VLUs	A	–	A	A
Ulcer healing (%) vs control group (*P* value)	61.3% vs 47.7% (*P* = .03)	–	63.1% vs 38.3%	49.4% vs 29.8%
RR (CI)	1.36 (95% CI, 1.07-1.74)	C	(*P* < .0001)	(*P* < .0001)
Time (weeks) to healing vs control (*P* value)	16 vs 21 (*P* = .0034)	1.70 (95% CI, 1.24-2.34)	1.70 (95% CI, 1.30-2.24)	1.70 (95% CI, 1.33-2.17)
No. of studies (No. of patients)	5 (723)	3 (279)	11 (864)	3 (438)

*CI,* Confidence interval; *HR,* hydroxyethylrutosides; *MPFF,* micronized purified flavonoid fraction; *RR,* relative risk.

A, B and C: level of scientific evidence.

#### MPFF

MPFF acts on several factors important for the venous homeostasis maintenance, as well as for VLU healing, such as venous tone and lymphatic drainage increase, inflammation reduction, vessel permeability and free radical generation decrease. In the meta-analysis of 5 prospective RCTs with 723 VLU patients, the efficacy of the standard treatment (medical compression plus local wound care) together with MPFF was compared with standard treatment (three trials) or standard treatment plus placebo. The duration of the treatment was 2 months in one trial (500 mg twice daily) or 6 months in the other four trials. In an analysis of the four trials that included 616 patients who continued MPFF for 6 months, 61.3% of the patients in the MPFF were completely healed vs 47.7% in the control group (relative risk reduction [RRR], 32%; 95% CI, 3%-70%). This difference was associated with shorter healing time (16 weeks vs 21 weeks). Also, in the analysis after 2 months, which included 5 RCTs with 723 patients available, a significant higher chance for ulcer healing was noticed in the group with adjunctive MPFF treatment (*P* = .015). The benefits of MPFF were noticed especially in patients with an ulcer size between 5 and 10 cm^2^ (RRR, 40%; 95% CI, 6%-87%) and with a duration 6 to 12 months (RRR, 44%; 95%, CI 6%-97%). In the ulcers larger than 10 cm^2^ or smaller than 5 cm^2^, no significant effect of MPFF over standard treatment was found.^54^

The MPFF benefits in VLU treatment were confirmed other reviews as well. In the Cochrane meta-analysis focusing on the flavonoids in the treatment of the VLU and five clinical trials with MPFF, a higher rate of healing of VLUs was reported in MPFF group than in the control one (RR, 1.36; 95% CI, 1.07-1.74).^55^

#### Sulodexide

Sulodexide is a glycosaminoglycan and represents another pluripotential VAC that can have a beneficial effect on CVD-related pathological processes and VLU healing. In this respect, sulodexide has strong anti-inflammatory activity, and vessel wall permeability decreases as well as free radical generation. An influence on metalloproteinase expression and activity seems to be especially interesting.^26^ In the SUAVIS placebo-controlled RCT with 235 patients randomized, the use of sulodexide (together with compression and local treatment) resulted in a complete 2-month ulcer healing rate of 35% in the sulodexide group and 20.9% in the placebo group (*P* = .018). The 3-month VLU healing rate was 52.5% in the sulodexide group vs 32.7% in the placebo group (*P* = .004).^56^

Kucharzewski et al^57^ added sulodexide to standard therapy and noticed complete healing of VLUs after 7 weeks in 70% of the patients, vs 35% in the group with standard therapy only. According to a Cochrane analysis including three RCTs with 438 patients, the use of sulodexide in addition to the standard treatment improves VLU healing from 29.8% to 49.4% (RR, 1.66; 95% CI, 1.30-2.12).^58^ Another meta-analysis performed on four trials and 482 randomized patients suggests significant improvement in the ulcer healing when adding sulodexide to the standard therapy (RR, 1.70; 95% CI, 1.33-2.17).^59^ González Ochoa^60^ in a nonrandomized prospective study on the VLU patient cohort, documented that the combined treatment with sulodexide and MPFF was more effective in accelerating ulcer healing and controlling pain, than the use of MPFF alone (in both groups the standard treatment including compression was applied).

#### HRs

The evidence concerning the efficacy of HRs is based on limited data and small RCTs. In the systematic review concerning the efficacy of HR for the improvement of the signs and symptoms of chronic venous insufficiency four trials focusing on the VLU patients were included.^32^ In two placebo-controlled trials (HR + compression vs placebo + compression treatment) and in one study comparing compression + HR vs compression alone, no additional benefit of the HR treatment in term of the ulcer healing was reported.^61^^,^^62^ The fourth trial which compared troxerutin vs placebo in VLU treatment (with compression use in both groups) found a statistically significant difference with the better outcomes in the troxerutin group (odds ratio, 2.91; 95% CI, 1.36-6.2).^63^

In a Cochrane analysis of flavonoids for treating VLUs, nine studies including four with HR were reported.^55^ The authors of this analysis emphasized the fact of the limited quality of the available data and an unclear risk of bias. Pooling three of these four trials (279 patients), the favorable effect of HR with respect to the number of ulcers healed was identified (RR, 1.70; 95% CI, 1.24-2.34).^55^

#### Pentoxifylline

Pentoxifylline is a xanthine derivative with a pleomorphic effect.^64^ The beneficial role of pentoxifylline was the subject of the several trials performed on the venous as well as mixed (venous and arterial) ulceration showing significant healing improvement. A 2012 Cochrane Review identified 11 trials involving 864 patients that compared pentoxifylline with placebo or no treatment.^65^ Pentoxifylline was more effective than placebo in terms of the complete VLU healing or significant improvement (RR, 1.7; 95% CI, 1.30-2.24) and was more effective than placebo with compression (RR, 1.56; 95% CI, 1.14-2.13). This translates to a NNT of 4.3 (95% CI, 3.3-6.4). The beneficial effects of pentoxifylline vs placebo was confirmed in the absence of medical compression in both study groups (RR, 2.25; 95% CI, 1.49-3.39).^65^ The level of evidence was high (grade A). Adverse effects, mostly gastrointestinal, were reported in 19.5% of patients receiving pentoxifylline and in 11.3% on placebo (RR, 1.56; 95% CI, 1.10-2.22).

#### Effect of VACs on skin changes

In contrast with the available evidence concerning VLU patients, the data concerning the clinical proof of the efficacy of VACs for skin changes improvement remains very limited.^14^ Despite the theoretical background, which suggests the potential efficacy of VACs in the inflammation control, the difficulties in the skin changes assessment as clinical outcome, as well as high heterogenicity of the CVD populations should be mentioned. The limited evidence suggests the efficacy of MPFF in leg redness and skin changes treatment related to CVD.^66^^,^^67^ According to sulodexide studies, the use of glycosaminoglycans can have beneficial effects also in the group of lipodermatosclerosis patients.^60^ Well-designed studies are needed to identify the group of the patients which benefits most from the VAC therapy in term of skin changes improvement. The compounds' selection as well as their dosage should also be studied for treatment of the variety of skin abnormalities observed in CVD patients. Currently, the evidence-based recommendations in this field remain very limited or not available.

## Conclusions

There is now considerable evidence that supports the use of VACs in patients with CVD. The evidence is strong for symptoms, edema, as well as for the healing of leg ulcers. Symptoms are subjective and investigations on the efficacy of VACs need blinded, placebo-controlled RCTs if the problem of the placebo effect is to be avoided. The results of such RCTs are now available and together with systematic reviews and meta-analyses they have contributed to the development of levels of evidence (A and B) in recent guidelines^15^^,^^17^ for specific combinations of drugs and symptoms. Knowledge of the specific effect that individual drugs have on different symptoms improves the armamentarium of a physician and one's confidence in their use. VACs can be used at all stages of CVD. However, the importance of effective treatment of patients in CEAP class C0s is highlighted by the fact that approximately 20% of all patients consulting their general practitioner for any reason could be assigned to class C0s.^68^ This is a group of patients for whom there is no alternative treatment.

A number of caveats are associated with the use of VACs. One cannot always rely on the patient's skills to name symptoms, which are feelings variably expressed and with different intensities, and have different meanings in the minds of individual patients.^27^ In addition, words used to describe symptoms are influenced by cultural and linguistic factors. Symptoms are not specific to CVD. For these reasons, a physician needs great care and experience to interpret the patient's history and decide on the appropriate investigations to exclude conditions other than CVD that may be present or comorbid and responsible for the symptoms. Absence of such an approach may lead to misuse of VACs causing failures and eventual disrepute.

Signs such as edema or ulcers can be measured objectively and studies on efficacy of VACs have been easier to perform. The evidence that certain VACs are effective in reducing edema is strong. Also, the evidence that the combination of a VAC or combinations of VACs with compression in speeding up the rate of venous ulcer healing is also strong.

## Author Contributions

Conception and design: MG, SK, TU, JC, AN

Analysis and interpretation: Not applicable

Data collection: Not applicable

Writing the article: MG, SK, TU, JC, AN

Critical revision of the article: MG, SK, TU, JC, AN

Final approval of the article: MG, SK, TU, JC, AN

Statistical analysis: Not applicable

Obtained funding: Not applicable

Overall responsibility: MG

MG, SK, TU, JC, and AN contributed equally to the concept and preparation of the manuscript.

## Funding

None.

## Disclosures

M.G. is a Scientific Advisor to the VitasupportMD company. J.C. is a Founder of VitasupportMD company.
